# Rates of success in hearing and grafting in the perichondrium-preserved palisade island graft technique

**DOI:** 10.1016/j.bjorl.2019.09.009

**Published:** 2019-11-02

**Authors:** Selahattin Genç, Halil Erdem Özel, Erdem Altıparmak, Serdar Başer, Şaban Eyisaraç, Ferit Bayakır, Fatih Özdoğan

**Affiliations:** Health Sciences University, Kocaeli Derince Education and Research Hospital, Department of Otorhinolaryngology, Derince, Turkey

**Keywords:** Chronic, Otitis media, Tympanoplasty, Cartilage, Graft

## Abstract

**Introduction:**

Various graft materials have been used in the tympanoplasty technique. Cartilage grafts are being used increasingly in recent years.

**Objective:**

The aim of this study was to present the comparative outcomes of the perichondrium-preserved palisade island graft technique previously defined by ourselves.

**Methods:**

We retrospectively compared the hearing and graft success rates in 108 patients with chronic otitis media, who had undergone cartilage tympanoplasty, where both island and perichondrium-preserved palisade graft techniques were used.

**Results:**

The success rates among the study and the control groups with regard to graft take were 97% and 93%, respectively. No significant difference was observed between the groups with regard to the postoperative mean pure tone values, improvement in air-bone gaps and reduction in air-bone gaps to under 20 dB. However, better results were observed in the study group.

**Conclusion:**

The perichondrium-preserved palisade island graft technique is an easy method with high graft success rates and hearing outcomes.

## Introduction

Tympanoplasty is a reconstructive surgical method that repairs the tympanic membrane and bony structures in the middle ear in patients with chronic otitis media.^1^ Tympanoplasty was first described by Wullstein and Zoellner.^2^, ^3^ Various graft materials such as temporal muscle fascia, periosteum, perichondrium, vein, cartilage and fat have been used for repair of the tympanic membrane ever since.^4^ Cartilage grafts are generally preferred in draining ears, presence of eustachian tube dysfunction, adhesive otitis media, retraction pocket, perforations that include all or more than 50% of the tympanic membrane and revision cases. The palisade and island graft techniques are the two main techniques described in cartilage tympanoplasty.^5^ There is no consensus in the literature on which of these techniques yield better hearing and graft take rates.^1^ The palisade technique increases the flexibility of the graft and reduces its mass by reducing the mass effect. However, displacement of palisades is a complication that may be observed in the postoperative period. In the island graft technique, flexibility of the graft may be reduced via the mass effect in the presence of full thickness cartilage; if it is placed as a whole, and the hearing outcomes may be negatively affected.

The aim of this study was to evaluate the comparative outcomes of the perichondrium-preserved palisade island graft technique,^6^ which was previously defined by us, with regard to graft uptake and hearing outcomes.

## Methods

The study was approved by the Kocaeli University Medical School Ethics Committee (KÜ GOKAEK 2016/227). A total of 461 patients with chronic otitis media who had undergone the operation by the same surgeons between January 2013 and January 2017 were retrospectively evaluated. Patients with cholesteatoma or tympanosclerotic middle ear mucosa-related ossicle fixation and those who used partial or total ossicle replacement prosthesis due to ossicle defects were excluded from the study. Patients undergoing canal wall down tympanomastoidectomy, butterfly tympanoplasty, and intact canal wall tympanomastoidectomy due to chronic mucosal diseases or revision surgery, were also excluded. The study included 108 similar patients undergoing primary tympanoplasty, who had intact and mobile ossicles and no drainage for a minimum of 3 months. The study group included 65 patients undergoing the perichondrium-preserved palisade island graft technique.^6^

**Figure 1:**
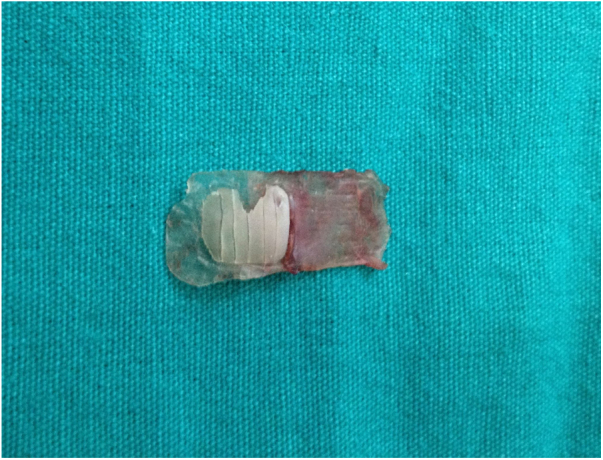
Perichondrium-preserved palisade island graft.

**Figure 2:**
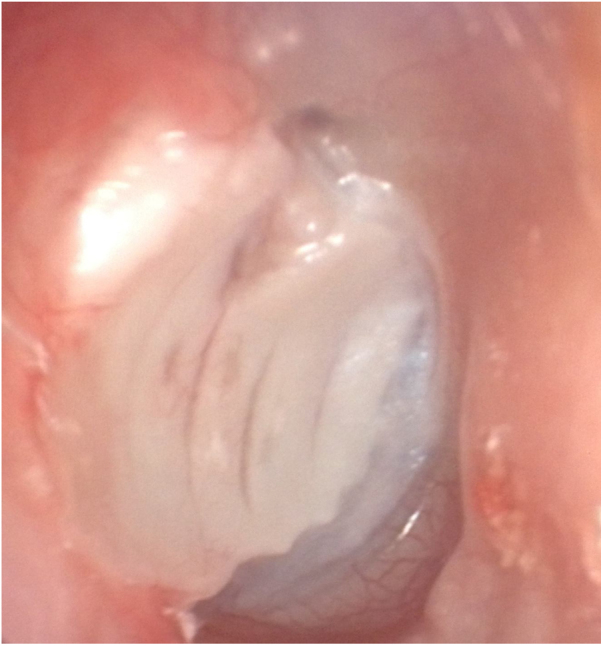
Postoperative first year appearance of the perichondrium-preserved palisade island graft.

**Figure 3:**
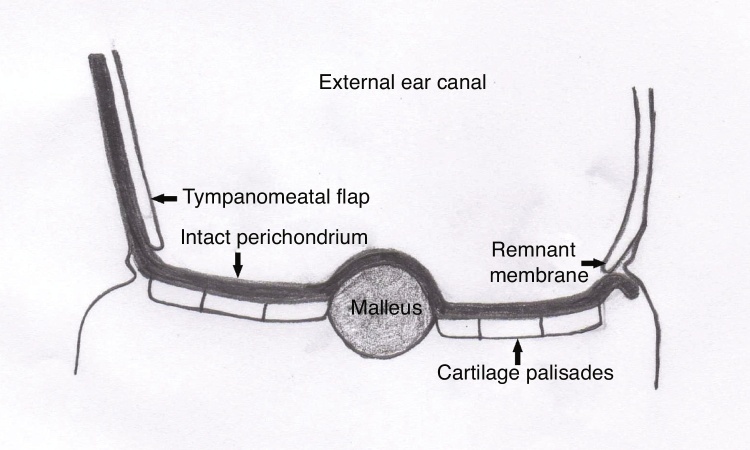
Graft placed with the underlay technique according to the tympanomeatal flap, fibrous anulus and membrane remnant, and the overlay technique according to the malleus. The intact perichondrium that keeps the palisades together faces the external ear canal.

The tragal cartilage is used as graft in this technique. The perichondrium over both surfaces of the cartilage is protected during graft harvesting. The perichondrium is elevated from the cartilage on one surface during graft preparation. The perichondrium on the other surface is kept non-elevated. The adjacent cartilage and the non-evelated perichondrium are protected and the cartilage is properly re-sized. The cartilage is thinned to a 0.5 mm thickness using a scalpel. The part of the cartilage block that corresponds to the proximal aspect of the manibrium mallei and lateral process of the malleus projection is partially resected by protecting the perichondrium underneath. Six palisades of approximately 1 mm size are formed on the cartilage block that are adjacent to the underlying perichondrium (Figure 1, Figure 2) and the cartilage graft is placed in such a position that the non-elevated intact perichondrium would face the external acoustic meatus. The graft is placed with the underlay technique according to the tympanomeatal flap, fibrous anulus and membrane remnant, and the overlay technique according to the malleus (Fig. 3). The graft was held in place supported by absorbable haemostatic gelfoam placed in the middle ear cavity. The control group included 43 patients in whom the island cartilage graft technique without cartilage thinning was used. The tragal cartilage was used as graft in this group. Mastoid dressings were removed on postoperative day 1 in the both groups. Oral antibiotic therapy was continued until postoperative day 7 and local treatment was begun with ofloxacine and corticosteroid drops onto the external auditory canal tract packing. External auditory canal tract packing was removed on postoperative day 10. Local treatment was continued at the same dose for two more weeks and gelfoam was cleared under otomicroscopy if it remained at the end of this duration.

The pre- and the post-operative sixth month data of the patients were obtained from the database including patient information; the data included detailed otomicroscopic examination findings and audiological evaluations. The site and size of the perforation on the tympanic membrane, whether the rest of the membrane was atrophic or not, retracted or sclerotic, and whether the middle ear mucosa was hypertrophic, sclerotic or wetter, were evaluated. The patients were evaluated with audiological tests performed in the preoperative period and at the postoperative 6^th^ month. Pure tone values at 250‒500‒1000‒2000‒4000 and 8000 Hz. frequencies were recorded. Bone and air conduction Pure Tone Averages (PTA) were determined using 500‒1000‒2000 Hz. hearing thresholds and the Air-Bone Gaps (ABG) were calculated. An ABG value of lower than 20 dB at the postoperative sixth month was evaluated as a successful outcome.

The data were analyzed using the software Statistical Product and Service Solutions (SPSS), Predictive Analytics SoftWare (PASW) and Statistics 21 (SPSS Inc.; IBM Corp., Chicago, IL, USA). The Student’s *t-*test and chi-squared test were applied to the measurements in all groups. A *p*-value of <0.05 was considered statistically significant.

## Results

The study and the control groups included 65 and 43 patients, respectively. Among the patients in the study group, 21 (32.3%) were male and 44 (67.7%) were female, and among the patients in the control group, 21 (48.8%) were male and 22 (51.2%) were female. The mean age of the patients was 33.4 years (range 9–63) in the study group and 32.1 years (range 11–64) in the control group. In the study group, 41 patients (63.1%) had undergone the operation on the right ear and 24 (39.9%) patients had undergone the operation on the left ear; these figures were 23 (53.5%) and 20 (46.5%) in the control group, respectively. The operation time was similar in the both groups. However, an additional time of approximately 5 min was required to prepare the graft in the palisade island graft technique. None of the patients in the study and control group had complications such as hematoma, infection and dizziness in the post-operative period. In both groups, postoperative first day pain was treated with paracetamol (administered parenterally in children) and tenoxicam in adult patients. After the first day, no significant complaints of pain were observed in either of the two groups.

The dimensions of the preoperative perforations were compared between the groups. In the study group, the dimensions of the perforation were less than half of the normal size of the membrane in 29.2% of the patients, and subtotal perforation was observed in 70.8%. In the control group, these rates were 27.9% and 72.1%, respectively. No significant difference was observed between the groups with regard to the preoperative dimensions of the perforation (*p* = 0.882).

The preoperative and postoperative air conduction Pure Tone Averages (PTA) observed in the study group were 34.19 ± 11.5 dB and 19.45 ± 8.2 dB, respectively. The preoperative and postoperative air conduction PTAs observed in the control group were 36.05 ± 14.6 dB and 22.09 ± 9.6 dB, respectively. The differences were statistically significant for both groups (*p* < 0.001). However, no significant difference was observed between the groups for either the preoperative or the postoperative values (*p* = 0.357 and *p* = 0.586, respectively).

The preoperative and postoperative bone conduction PTA values were determined as 9.21 ± 6.7 dB and 8.12 ± 6.1 dB in the study group. The difference was not significant (*p* = 0.110). The preoperative and postoperative bone conduction PTA values were determined as 8.62±7.1 dB and 6.61±5.6 dB in the control group. No significant difference was observed for this group either (*p* = 0.120). No significant difference was observed between the groups with regard to the preoperative or the postoperative values (*p* = 0.620 and *p* = 0.454, respectively).

In the study group, the mean Air-Bone Gaps (ABG) were 24.97 ± 9.9 dB in the preoperative period and 11.24 ± 5.6 dB in the postoperative period. The difference was statistically significant (*p* < 0.001). In the control group, ABGs were 27.41 ± 11.5 dB in the preoperative period and 15.62 ± 7.6 dB in the postoperative period. The difference was statistically significant (*p* < 0.001). No significant difference was observed between the groups with regard to preoperative and postoperative values (*p* = 0.595 and *p* = 0.140, respectively).

A closure below 20 dB in ABG was accepted as a successful outcome. The rates of AGB under 20 dB were 59/65 (90.8%) in the study group and 38/43 (88.4%) in the control group. The difference was statistically insignificant (*p* = 0.751).

The graft success rates were determined as 63/65 (97%) in the study group and 40/43 (93%) in the control group (Table 1).

**Table 1:** Patient characteristics in the study and the control groups.

Characteristics	Study Group Mean ± SD	Control Group Mean ± SD	*p*
Patient number	65	43	
Age	33.4	32.1	
Male patient	21	21	
Preoperative perforation size < 50%	19	12	0.882^a^
Subtotal perforation	46	31	0.882^a^
Famele patient	44	22	
Preoperative air conduction PTA (dB)	34.19 ± 11.5	36.05 ± 14.6	0.357^b^
Postoperative air conduction PTA (dB)	19.45 ± 8.2	22.09 ± 9.6	0.586^b^
Preoperative ABG (dB)	24.97 ± 9.9	27.41 ± 11.5	0.595^b^
Postoperative ABG (dB)	11.24 ± 5.6	15.62 ± 7.6	0.140^b^
Postoperative ABG (dB) < 20	59/65 (90.8%)	38/43 (88.4%)	0.751^a^
Graft take rate (%)	97%	93%	

SD, Standard Deviation; PTA, Pure Tone Average; ABG, Air-Bone Gap; dB, Decibel.

a Chi-square test.

b *t*-test.

## Discussion

Temporal muscle fascial graft and cartilage graft are the most frequently preferred graft materials in tympanoplasty. Cartilage grafts are particularly preferred in the presence of draining ears, eustachian tube dysfunction, adhesive otitis media, retraction pocket or tympanosclerosis.^7^, ^8^, ^9^ These grafts are preferred since they are substantially fed through diffusion, their metabolism is slow, and they are resistant to infections and retractions.^1^, ^8^, ^10^, ^11^, ^12^, ^13^, ^14^

Heermann^15^ was the first to describe the use of cartilage palisade grafts in tympanoplasty and the graft success rates have been reported starting from 94%. Subsequently, many techniques have been described by different authors. Tos has grouped cartilage tympanoplasty techniques under six titles.^5^ Two main cartilage grafts are used in these techniques. These are the palisade and the island graft techniques.

In the palisade graft technique, the palisades formed increase the flexibility of the graft, reduce it’s mass, and thereby, its mass effect. However, in this technique, postoperative displacement of the cartilage palisades may result in graft failure or worsened hearing. In the island graft technique, when the cartilage island is placed as whole-thickness and as a whole, the flexibility of the membrane may be reduced by the mass effect, and the hearing outcomes may be negatively affected. Considering these risks, we presented the “Perichondrium-preserved palisade island graft” technique for the first time in 2016.^6^ We used the palisade and island cartilage graft in the same technique. We thinned the graft we formed from the tragal cartilage by half, and sliced the cartilage block as in the palisade technique, thereby increasing the flexibility of the graft, and reduced its mass and related mass effect. We prevented displacement of the palisades by protecting the perichondrium on one side of the cartilage as in the island graft (Figure 1, Figure 2). Through this technique, we eliminated some disadvantages of the palisade and island techniques, and used their advantages in the same technique.

**Figure 4:**
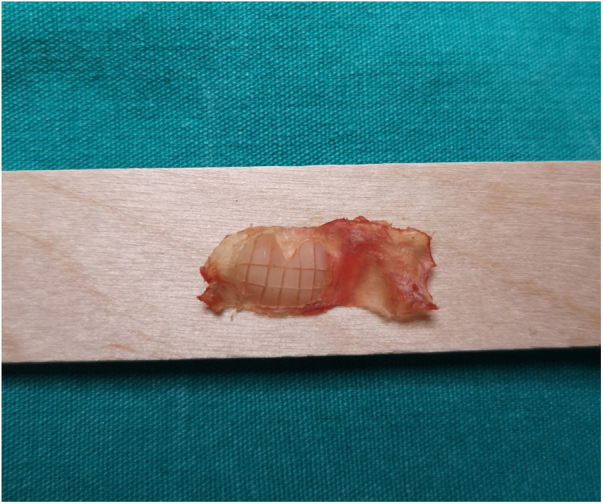
Curling may be prevented by turning the cartilage graft into a mosaic-like shape.

Different thickness measurements of the cartilage graft are encountered in the literature, considering its acoustic transfer property. Some authors use the cartilage graft as whole-layer such as that in the island technique, and some recommend using it after thinning.^9^, ^16^, ^17^, ^18^, ^19^, ^20^ In the perichondrium-preserved palisade island graft technique, we used the graft we formed from the tragal cartilage after thinning by half.^6^ We did not prefer to thin the cartilage further in order to avoid reducing the resistance of the graft while increasing its acoustic transfer property. The graft thickness we recommend is 0.5 mm, which shows a similar acoustic transfer property to the normal tympanic membrane. However, graft curling may be observed during the thinning procedure of the cartilage, which we observed in one patient. In this patient, we solved the problem by changing the graft into an island graft formed of mosaic-like, small, rectangular pieces of cartilage (Fig. 4). Curling appears to be a disadvantage of our technique; however, its rate is low and solving this problem is not difficult.

According to the hearing outcomes, the differences between the pre- and post-operative Air-Bone Gap (ABG) and Pure Tone Average (PTA) values were statistically significant in both the study and the control groups. However, the differences in the postoperative ABG and PTA values were not significant between the groups. The postoperative PTA was 19.45 dB in the study group, which was more successful. The postoperative PTA value was 22.09 in the control group. Postoperative ABG was 15.62 dB in the control group and 11.24 dB in the study group. The decrease in AGB below 20 dB was 90.8% successful in the study group, and the success rate was 88.4% in the control group. When the study group was compared with the literature, better results were seen to have been obtained than the hearing results of Kirazlı,^8^ Onal,^21^ Altuna,^22^ and Yetiser.^23^ Both the PTA outcomes and improvement in ABG demonstrate that the perichondrium-preserved palisade island graft technique is a successful technique.

Generally, the graft success rates vary between 71%–100% in the literature.^9^, ^15^, ^17^, ^21^, ^22^, ^23^, ^24^, ^25^, ^26^, ^27^, ^28^, ^29^, ^30^, ^31^ In the palisade cartilage tympanoplasty technique, the graft success rates range from 90%–100%.^7^, ^15^, ^28^, ^32^ In our study, the graft success rate was 97% in the study group. Compared to the work of Özbek^28^ and Vashishth^7^ using the palisade technique, the success rates of our study group were found to be more successful. These outcomes suggest that this technique is also more successful with regard to graft uptake.

## Conclusion

The perichondrium-preserved palisade island graft technique is an easy technique with high graft success rate and successful hearing outcomes.

## Conflicts of interest

The authors declare no conflicts of interest.
